# A case report of May-Thurner syndrome induced by anterior lumbar disc herniation: Novel treatment with radiofrequency thermocoagulation

**DOI:** 10.1097/MD.0000000000017706

**Published:** 2019-11-01

**Authors:** Feng Xu, Zhisen Tian, Xiuying Huang, Yipeng Xiang, Liyu Yao, Congcong Zou, Changfeng Fu, Yuanyi Wang

**Affiliations:** a Department of Spine Surgery, The First Hospital of Jilin University; b Department of Orthopedics, The China-Japan Union Hospital of Jilin University; c Second Operating Room; d Department of Pediatrics Surgery, The First Hospital of Jilin University, Changchun, Jilin, China.

**Keywords:** anterior lumbar disc herniation, inferior vena cava stenosis, lumbar degeneration-related May-Thurner syndrome, radiofrequency thermocoagulation

## Abstract

**Rationale:**

Lumbar degeneration-related May-Thurner syndrome (dMTS) is characterized by venous compression induced by degenerated lower lumbar structures. Treatment strategies for May-Thurner syndrome (MTS) include clearing the thrombus and correcting venous compression. Despite having different etiological factors from other MTS types, treatments for dMTS are similar, including endovascular angioplasty and continuous anticoagulation therapies. Thus, a particular treatment was designed herein to focus on compressive lumbar structures instead of intravenous management.

**Patient concerns:**

A 59-year-old female patient with dMTS, which was induced by inferior vena cava (IVC) stenosis compressed by L4-5 anterior disc herniation.

**Diagnosis:**

The patient was diagnosed with IVC stenosis and L4-5 lumbar disc herniation based on abdominal computed tomography, ultrasound, and lumbar magnetic resonance imaging findings.

**Interventions:**

Radiofrequency thermocoagulation (RF) was applied to the patient to decrease the compression caused by anterior disc herniation.

**Outcomes:**

After surgery, the patient's swelling started to improve within 5 hours and completely diminished after 48 hours. Postsurgical abdominal ultrasound showed that her IVC patency increased by 20%. On follow-up, her leg symptoms did not recur at 12 months after surgery.

**Lessons:**

We provided a novel idea in the treatment of dMTS, in which we shifted the treatment focus from endovascular patency restoration to extravascular decompression. Our case proved that RF was effective in treating dMTS, which is a complementary treatment modality to angioplasty.

## 1 Introduction

May-Thurner syndrome (MTS) is a rare vascular condition caused by narrowing of the vein and was first described in 1950 by Dr May and Dr Thurner. They systemically analyzed the histology of the intravenous “spur” and categorized it into 3 subtypes according to their morphologies.^[1]^ Severe compression of the iliac vein or inferior vena cava (IVC) may lead to multiple sequalae, including deep vein thrombosis (DVT), pulmonary emboli, and different symptoms such as pain, heaviness, and swelling in the lower extremities. MTS has been classified into 3 categories according to the distinct patho-anatomy and morphology observed in computed tomography (CT) images, among which lumbar degeneration-related MTS (dMTS) is caused by the compressive effect of degenerated lower lumbar structures.^[2]^ Patients with dMTS have a later age of onset because the main initiating factor is lumbar degeneration, which mostly occurs among the elderly population. Current treatment of MTS consists of endovascular management of thrombolysis and angioplasty, which results in excellent immediate and short-term outcomes and satisfying long-term outcomes.^[3]^ However, as a secondary change induced by degenerated lumbar structures, dMTS has different etiological factors when compared with other MTS types. Hence, extravascular decompression targeting the compressive lumbar structure seems to be another reasonable treatment.

Here, we report the use of radiofrequency thermocoagulation (RF) to treat a patient with dMTS, who was recurrence-free at the 12-month follow-up visit, and we believe that this case provides a novel idea in the treatment of dMTS.

## 2 Case report

A 59-year-old female patient with a long history of low back pain presented with swelling and distending pain in her lower extremities for 1 month; all her symptoms were aggravated after overexertion and improved with rest. Her lower limb symptoms were less severe in the morning and worse at night; however, since 1 week before the presentation, the swelling seldom resolved.

On physical examination, the patient showed stable vital features. During the walking test, the patient presented with intermittent claudication, which was more prominent on the left side. Her left thigh perimeter was 59 cm in the morning and 63 cm in the evening, while the right thigh perimeter was 55 to 59 cm. On examination of the spine, the patient had percussion pain and tenderness on the L4 and L5 spinous processes and in the corresponding paraspinal muscle. The distending pain in the calf and heaviness appeared after walking or standing for 200 m or for 5 minutes. In the lying position, there were no remarkable neurological symptoms, but there was a slight loss of key muscle force and pitting edema over the lower extremities. According to the visual analog scale (VAS), the patient scored 6 for her low back pain and 4 for her calf pain.

**Figure 1:**
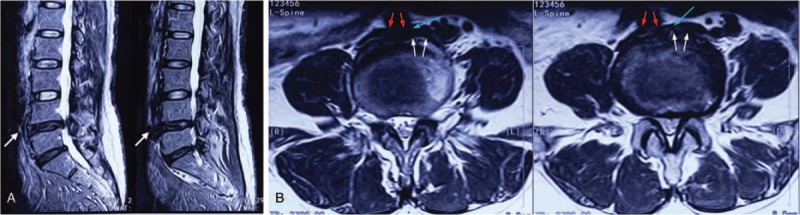
Preoperative MRI shows the lumbar disc degeneration, and the white arrows point out the up-migrating anterior LDH on the segment of L4-5 and the disc fragments enlarged the front margin of L4-5 disc (A). Transverse MRI plain shows irregular front margins at the level of L4-5 intervertebral disc and inferior vena cava (pointed by blue arrow) under the compressive effect of anterior LDH (white arrow) and the overlying right iliac common artery (red arrow) (B). LDH = lumbar disc herniation, MRI = magnetic resonance imaging.

**Figure 2:**
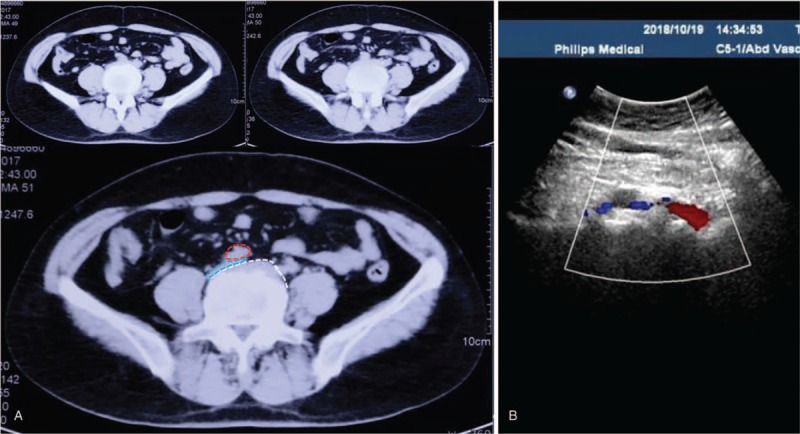
Preoperative CT shows the anterior LDH on the segment of L4-5, which causes the stenosis of inferior vena cava (A). The right iliac common artery is outlined by red, the LDH is outlined in white, and the compressed inferior vena cava is outlined in blue. Preoperative abdomen Doppler ultrasound shows the over 50% patency loss on the left inferior vena cava (B). CT = computed tomography, LDH = lumbar disc herniation.

Laboratory tests and electrocardiograph demonstrated normal renal and cardiac functioning, which excluded the probability of renal or cardiac edema. Lower extremity ultrasound showed no DVT or venous obstruction. To identify the etiology of her low back pain, lumbar magnetic resonance imaging (MRI) was conducted, which revealed lumbar disc degeneration (LDD) and anterior lumbar disc herniation (LDH) in the segment L4-5 (Fig. 1). Furthermore, the narrowed IVC was visualized in the space between the anterior protrusion and right common iliac artery, both on the MRI and abdominal CT (Figs. 1C, D and 2A–C). An abdominal Doppler ultrasound indicated over 50% patency loss of the IVC due to the compression induced by the anterior LDH (Fig. 2D). Further examinations such as a venogram and CT with contrast were rejected due to the invasiveness and the patient's refusal of the contrast agent. Based on the current results, this patient was diagnosed with L4-5 lumbar disc anterior herniation, moderate IVC stenosis, and dMTS.

**Figure 3:**
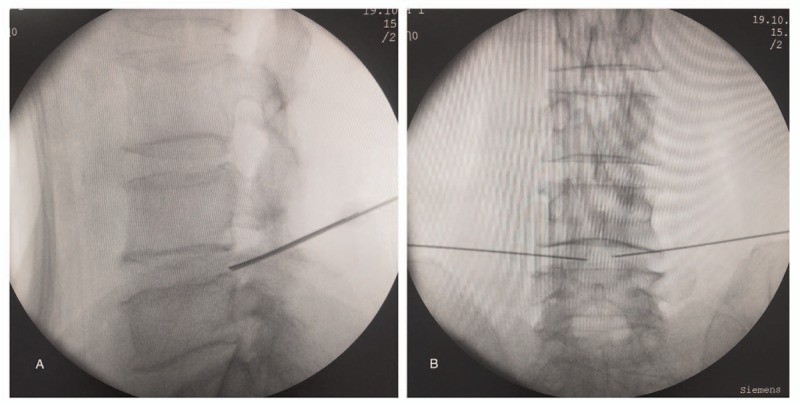
C-arm fluoroscopy monitored lumbar disc radiofrequency thermocoagulation. The puncture needles were inserted bilaterally and symmetrically until the needles reached the edge of the Kambin triangle (A). The puncture needles were further inserted through the annulus fibrosus into the disc towards the midline on the anteroposterior view (B).

For surgery, we aimed the treatment at the anterior LDH instead of endovascular management because of the severe IVC compression of the anterior LDH and the evidence against DVT. RF was adopted instead of conventional intravenous stents. The patient was settled in a prone position, and the L4-5 disc and 2 puncture points were confirmed via C-arm fluoroscopy. After routine disinfection and sterile towel placement, local anesthesia was applied. Monitored by C-arm fluoroscopy, the puncture needles were inserted bilaterally and symmetrically until the needles reached the edge of the Kambin triangle (Fig. 3A). After confirming that the needles were in the correct direction, they were further inserted through the annulus fibrosus into the nucleus pulposus towards the midline, on the anteroposterior view (Fig. 3B). Then, the monopolar RF electrode was placed through the puncture needle to the central part of the disc complex. After confirming the absence of nerve irritation, thermocoagulation was applied at 50 °C, 60 °C, and 70 °C for 60 seconds each, followed by 80 °C and 90 °C for 90 seconds each without rest between temperature changes. During RF thermocoagulation, the patient reported only warmth at the site of her low back pain, and no burning feeling on either lower extremity. The total operation time was less than 30 minutes, and blood loss was minimal.

**Figure 4:**
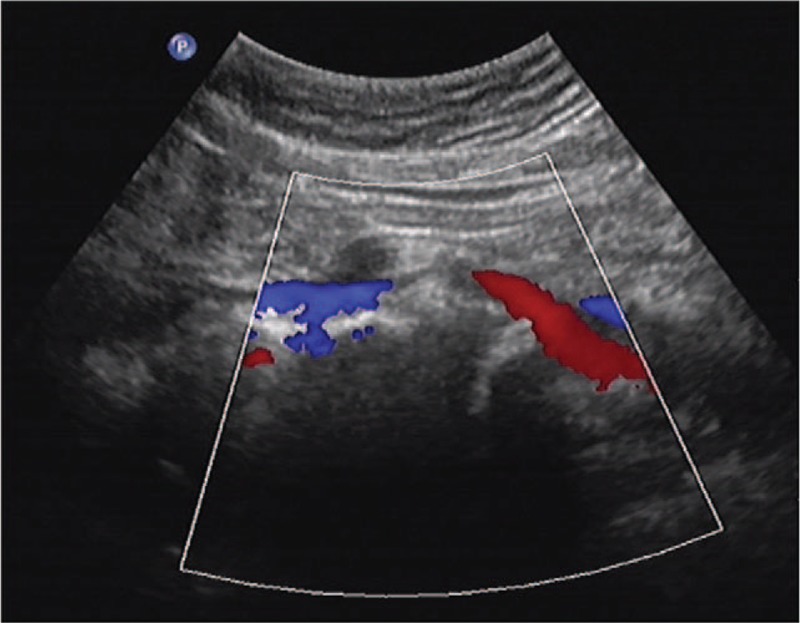
Postoperative abdomen Doppler shows that as the stenosis of inferior vena cava was relieved, the venous patency increased to 70%.

After the procedure, the patient's swelling on both extremities started to relieve within 5 hours, and it completely diminished after 48 hours. Her VAS decreased to 1 and 0 at her lower back and calves, respectively. Postoperative abdominal ultrasound confirmed that the venous patency of the left common iliac vein increased to 70%, which represented mild venous stenosis (Fig. 4). On follow up, only occasional slight lower back pain was reported, and her leg symptoms did not recur at 12 months after surgery.

## 3 Discussion

MTS is defined as venous compression caused by the arterial system against the extrinsic bony structure.^[4]^ To be specific, the anatomical variation of the involved artery and lower lumbar vertebral body may provide a “squeezing” effect, which mostly affects the left common iliac vein that runs in the space between the vertebral body and right common iliac artery.^[5]^ In addition, MTS affects the left common iliac vein; compression of the common iliac vein of either side by different arteries have also been reported.^[6,7]^ In this case, the implicated vein was the IVC, which is a rare variant of MTS, first reported by Fretz et al^[8]^ Together with our current case, the knowledge on the compressing range of MTS has been broadened.

MTS occurs predominantly among females in their twenties or thirties, and it has been well reported among the elderly.^[9]^ Kibbe et al reported that up to two-thirds of the asymptomatic patients had left common iliac vein compression of greater than 25%, and almost one-fourth had over 50% compression, which not only revealed the high prevalence of the May-Thurner anatomy, but it also suggests that this might be a common pattern.^[10]^ In spite of the high prevalence of venous stenosis among the population, only a few cases (3%–5%) come with sequelae, as most MTS patients are asymptomatic. Acute or gradual symptoms such as swelling, tenderness, or paresthesia of the lower extremities after prolonged sitting or standing might appear as the disease develops.^[7]^ Meanwhile, venous thrombosis in various locations might secondarily take place along with the progression of MTS, such as DVT, chronic venous insufficiency, venous rupture, and severe complications caused by migrating emboli such as a stroke or transient ischemic attack.^[4,11,12]^

In this case, the patient's main complaints were swelling and moderate pain in the lower extremities. The patient underwent Doppler ultrasound, abdominal CT, and lumbar MRI, as these noninvasive examinations are effective in detecting venous compression and revealing the anatomical characteristics of MTS. However, the gold standard for MTS diagnosis is venography,^[13]^ which was rejected by the patient. According to the results of the imaging tests, the etiological factor was not only the overlying right iliac common artery, but also the occupancy effect of severe anterior herniation of the L4-5 intervertebral disc.

LDD is a common cause of low back pain and neural disorders, but complications of circulation are rarely documented. Ou-Yang et al have classified MTS according to CT findings as follows: simple MTS, dMTS, and other-cause MTS. Among these, dMTS is caused by the compression of the artery in the ventral side, together with the space-occupying the herniated lumbar disc or proliferated bony structures at the back, with the development of LDD. Accordingly, lumbar degenerative changes such as anterior disc herniation, spondylolisthesis, osteophyte, and ligament ossification in the lower lumbar segments may lead to venous compression.

While the treatment of MTS has evolved rapidly within recent years, the basic strategy for treating MTS remains the same: restore blood flow and get rid of the thrombosis caused by the narrowed common iliac vein.^[5]^ To be specific, the classic invasive and continuous treatment includes thrombolysis, angioplasty, and anticoagulation therapies. Stenting is the contemporary treatment modality for MTS patients presenting with an indication for operation, and it is effective for the restoration patency. The patency rate approaches 100% within the first year, which is superior to that from conventional surgery.^[14,15]^ Jayaraj et al found that all degrees of severity of MTS resulted in symptomatic relief after stenting, whereas the cohort that had over 90% compression may relapse within 2 years after surgery.^[16]^ Despite the short-term complications such as bleeding and hematoma that are decreased under the guidance of ultrasound,^[17]^ elevating evidence of long-term complications such as re-thrombosis, clotting off, and stent crisis are documented.^[18]^ Anticoagulation therapy is routinely administered postoperatively, although the re-thrombosis rate has been significantly reduced by the combined application of stenting and anticoagulation therapy^[19]^; thus, the recurrence rate among the severely affected patients was still remarkable.^[16]^ Stent complications, including in-stent stenosis and stent occlusion, are the most frequently seen postoperative incidences, requiring stent revision or operation if severe symptoms appear. The most dangerous stent crises are stent migration and vein rupture. Most migrating stents stay in the venous vasculature, which results in mild or no symptoms, and requires expectant treatment.^[20]^ However, when a more concerning presentation occurs, such as heart or lung migration, further procedures are necessary to retrieve the stent.^[18,21]^

When compared to the other types of MTS, a dMTS patient has normal vascular anatomy until the appearance of compression induced by the solid occupancy effect from the degenerated lumbar structures. To our knowledge, current treatment strategies for dMTS aim at endovascular management similar to that accepted for the other MTS types, in which angioplasty, including balloon distraction and stenting, is applied to confront the extravascular compression.^[2,22]^ We present a novel approach for the treatment of dMTS by targeting the compressive lumbar structure instead of performing angioplasty, and we achieved a satisfying outcome. The decision regarding the technique was determined by the specific anatomy of dMTS. RF is a minimally invasive intervention method for intervertebral discoplasty, and it acts by breaking down the collagen and reducing the intradiscal pressure. Recently, RF has fallen out of popularity of use due to the rapid development of other minimally invasive surgeries. However, RF is still an effective procedure for relieving discogenic symptoms. By minimizing intradiscal pressure, RF significantly improved venous patency with no recurrence at the 1-year follow-up. The advantages of our strategy are:

(1) no implant is used, which avoids complications;
(2) and no long-term postoperative therapy is needed.

However, with regard to the experience of the application of lumbar disc RF, the drawbacks of the treatment were predictable: first, the recurrence rate might be relatively high. According to the report on the complications of similar application of RF in LDH treatment, the recurrence rate is 4.7% to 11.5% within the first 2 years.^[23]^ Second, the unclear decompression may lead to remission failure, and in this situation, surgery with precise outcome such as endoscopic lumbar discectomy is required. Third, surgical complications such as nerve root injury might occur. Finally, the indication of this method is restricted to dMTS with protruding intervertebral disc and without femoral or popliteal vein thrombosis extension. In this case, the venous patency was increased from 50% to 70% by RF, which turned moderate stenosis to mild. However, the relieving effect of RF alone is not sufficient in severe dMTS. In this condition, the combination of venous stent and RF might be a solution, where the stent assures restoration of patency and RF prevents stenting failure caused by progressive LDD.

## 4 Conclusion

Collectively, our case provided a new treatment approach for dMTS, in which we transferred the treatment focus from endovascular patency restoration to extravascular decompression. However, further investigation such as long-term follow-up is warranted. Our case proved that RF was effective in treating dMTS, which is a complementary treatment modality to angioplasty.

## Author contributions

**Conceptualization:** Changfeng Fu, Yuanyi Wang.

**Funding acquisition:** Yuanyi Wang.

**Investigation:** Yipeng Xiang, Yuanyi Wang.

**Project administration:** Yuanyi Wang.

**Resources:** Xiuying Huang.

**Software:** Liyu Yao.

**Visualization:** Liyu Yao, Congcong Zou.

**Writing – original draft:** Feng Xu, Zhisen Tian, Yuanyi Wang.

**Writing – review and editing:** Yuanyi Wang.
